# Thermolabile drug storage in an ambulatory setting

**DOI:** 10.1038/s41598-021-85413-0

**Published:** 2021-03-16

**Authors:** Fernando do Pazo-Oubiña, Bartomeu Alorda-Ladaria, Ana Gomez-Lobon, Bàrbara Boyeras-Vallespir, María Margalida Santandreu-Estelrich, Clara Martorell-Puigserver, María Gomez-Zamora, Pere Ventayol-Bosch, Olga Delgado-Sanchez

**Affiliations:** 1 Pharmacy Department, Son Espases University Hospital, Cra. de Valldemossa, 79, 07120 Palma, Illes Balears Spain; 2 eHealth and Telemedicine Research Group, IdisBa, Cra. Valldemossa, Palma, Illes Balears Spain; 3 Engineering of Industrial and Building Department, University of the Balearic Islands, Ctra. Valldemossa, km. 7,5., Palma, Illes Balears Spain

**Keywords:** Drug regulation, Therapeutics

## Abstract

More thermolabile drugs are becoming available, and in most cases, these medications are dispensed to ambulatory patients. However, there is no regulation once medications are dispensed to patients and little is known with regard to what happens during transport and home storage. Previous studies suggest that these drugs are improperly stored. The present study was designed to determine the storage conditions of thermolabile drugs once they are dispensed to the patient in the Hospital Pharmacy Department. This is a prospective observational study to assess the temperature profile of 7 thermolabile drugs once they are dispensed to ambulatory patients at a tertiary care hospital. A data logger was added to the medication packaging. Temperature was considered inappropriate if one of the following circumstances were met: any temperature record less than or equal to 0 °C or over 25 °C; temperatures between 0–2 or 8–25 °C for a continuous period over 30 min. The time series of temperature measurements obtained from each data logger were analyzed as statistically independent variables. The data shown did not undergo any statistical treatment and must be considered directly related to thermal measurements. One hundred and fourteen patients were included and 107 patients were available for the analysis. On the whole, a mean of 50.6 days (SD 18.3) were measured and the mean temperature was 6.88 °C (SD 2.93). Three data loggers (2.8%) maintained all the measurements between 2 and 8 °C with less than 3 continuous data (< 30 min) out of this range but no data over 25 °C or below or equal to 0 °C. 28 (26.2%) data loggers had at least one measurement below zero, 1 data logger had a measurement greater than 25 °C and 75 (70.1%) were between 0 and 2 °C and/or between 8 and 25 °C for more than 30 min. In conclusion, once dispensed to patients, most thermolabile drugs are improperly stored. Future studies should focus on clinical consequences and possible solutions.

## Introduction

The World Health Organization defines temperature–sensitive pharmaceutical products as “any pharmaceutical good or product which, when not stored or transported within predefined environmental conditions and/or within predefined time limits, is degraded to the extent that it no longer performs as originally intended”^1^. In practice, temperature–sensitive drugs or thermolabile drugs (TD) are mainly represented by those that must be stored in a refrigerator within a temperature range between 2 and 8 °C. Most of these drugs are biological agents and changes in temperature may alter their structure and activity, so it is very important not to break the cold chain from their manufacture to the moment of administration to the patient, in order to ensure their effectiveness and safety.

In Europe, TD storage and distribution from the manufacturer to pharmacies is regulated by the Guidelines of 5 November 2013 on Good Distribution Practice of medicinal products for human use^2^.

However, there is no regulation once medications are dispensed to patients, and little is known with regard to what happens during transport and home storage. The medication leaves the professional circuit, and transportation and home storage may not ensure an adequate temperature range as recommended in the Summary of Product Characteristics.

More thermolabile drugs are becoming available, and they usually represent a high cost and are indicated in serious pathologies. In most cases, these medications are dispensed to ambulatory patients, making it necessary to extend the responsibility for conservation beyond what is done in the professional facilities, so as to guarantee adequate conservation in the patient’s home as well.

There are few data on the temperature conditions in which TD are stored once dispensed to patients. Two studies conducted in Spain showed that at least half of patients did not maintain an acceptable mean temperature during transport and home storage^3,4^. Using a stricter definition of adequate conditions of storage, a publication on biologic disease-modifying antirheumatic drugs found that only 6.7% of patients had stored their medications within the recommended temperature range^5^; while a similar study on golimumab showed that only 11.6% of injectors had been correctly stored^6^. A more recent study that focused on the storage of insulin in domestic refrigerators revealed that all the temperature logs were out of range (2 °C to 8 °C) at some point^7^. Although the definitions and results of adequate storage differ between previous studies, our hypothesis is that, once dispensed, thermolabile drugs are not properly preserved.

In Spain, the Spanish Agency of Medicines and Medical Devices establishes where drugs must be dispensed, and hospital use and hospital diagnosis drugs are mainly dispensed at the outpatient units in Hospital Pharmacy Departments. These units play a key role in dispensing medications to a high volume of patients every year.

The present study was designed to determine the storage conditions of thermolabile drugs by measuring the thermal profile once they are dispensed to the patient in the Hospital Pharmacy Department.

## Results

One hundred and fourteen patients were included. Seven patients did not return the logger and one did not fill in the questionnaire, so 107 patients were available for analysis. Median age (interquartile range, range) was 53 (40.5 to 67.5, 18 to 89) years of age and 61.7% were females.

Distribution of TD were as follows: adalimumab, 38 (35.6%); etanercept, 23 (21.5%); erythropoietin, 23 (21.5%); certolizumab pegol, 7 (6.5%); peginterferon alfa 2-a, 7 (6.5%); trametinib, 5 (4.7%); and darbepoetin alfa, 4 (3.7%).

**Figure 1:**
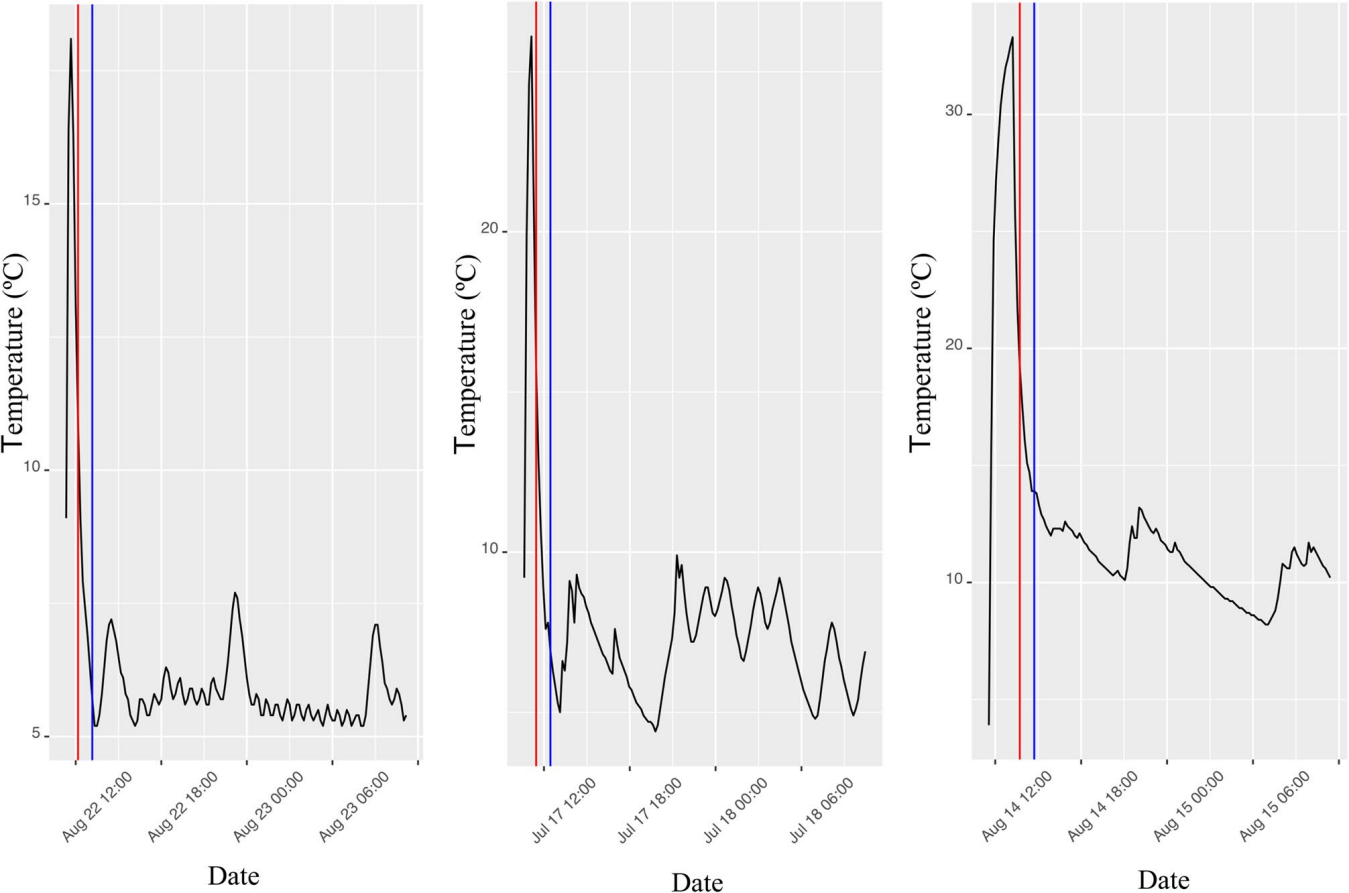
Examples of temperature variations during the first 24 h. Red line indicates the change detected by the algorithm. Blue line is red line plus 1 h and indicates the time to differentiate between transport and home storage.

By applying the algorithm explained in the Methods section, it was possible to differentiate the transport period for each data logger. Figure 1 shows some examples as to how the change was detected by analysing variations during the first 24 h of measurement.

**Figure 2:**
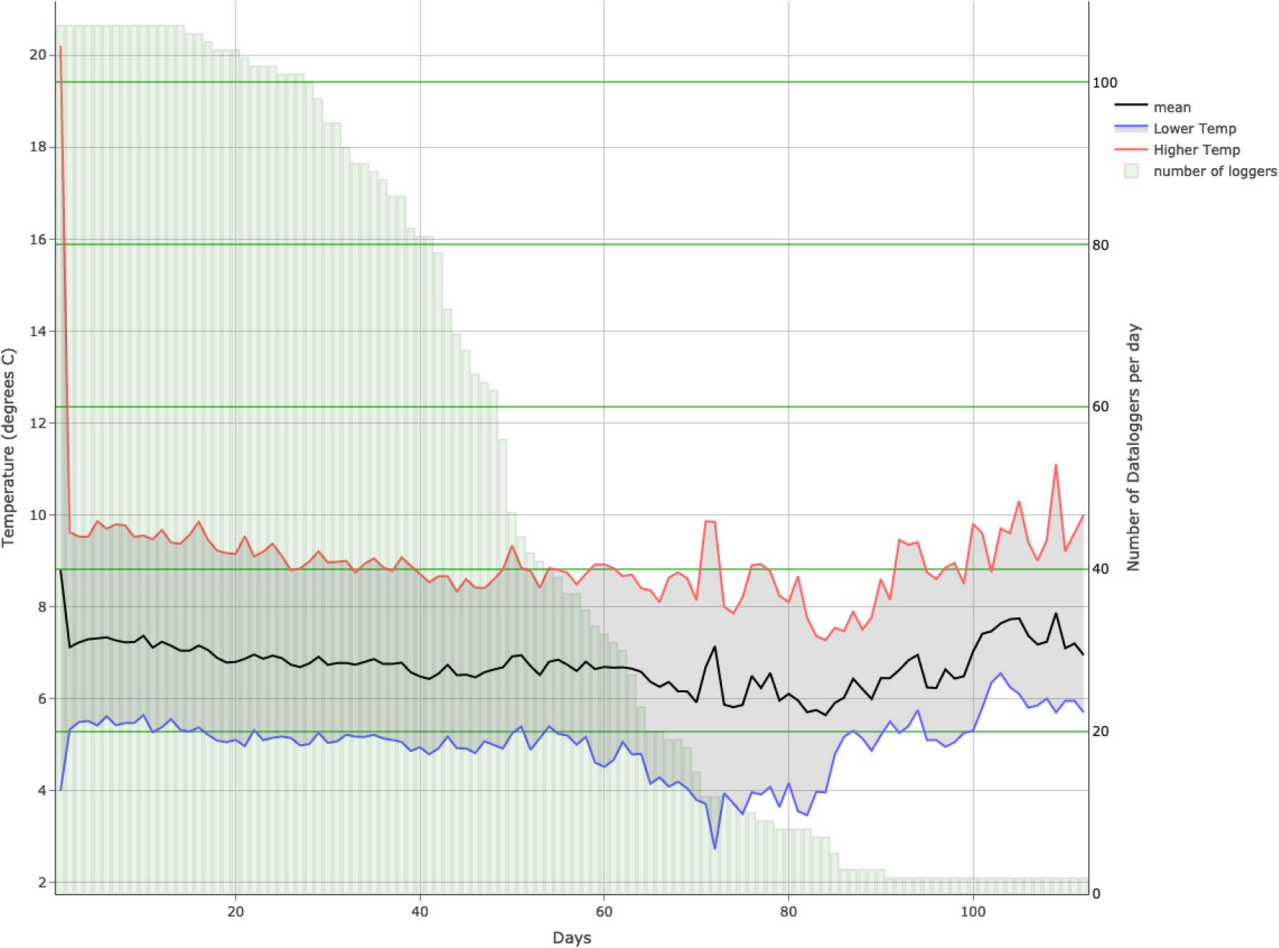
Daily evolution of temperatures and daily number of data loggers measuring data.

On the whole, a mean of 50.6 days (SD 18.3) were measured and the mean temperature was 6.88 °C (SD 2.93). Transportation took a mean time of 3.8 h (SD 6.7) with a mean temperature of 9.89 °C (SD 7.21). Home storage had a mean temperature of 6.86 °C (SD 2.89) over 50.4 days (SD 18.3). Figure 2 shows the evolution of daily mean, mean of the lowest, and mean of the highest temperatures, and the number of data loggers measuring data.

**Figure 3:**
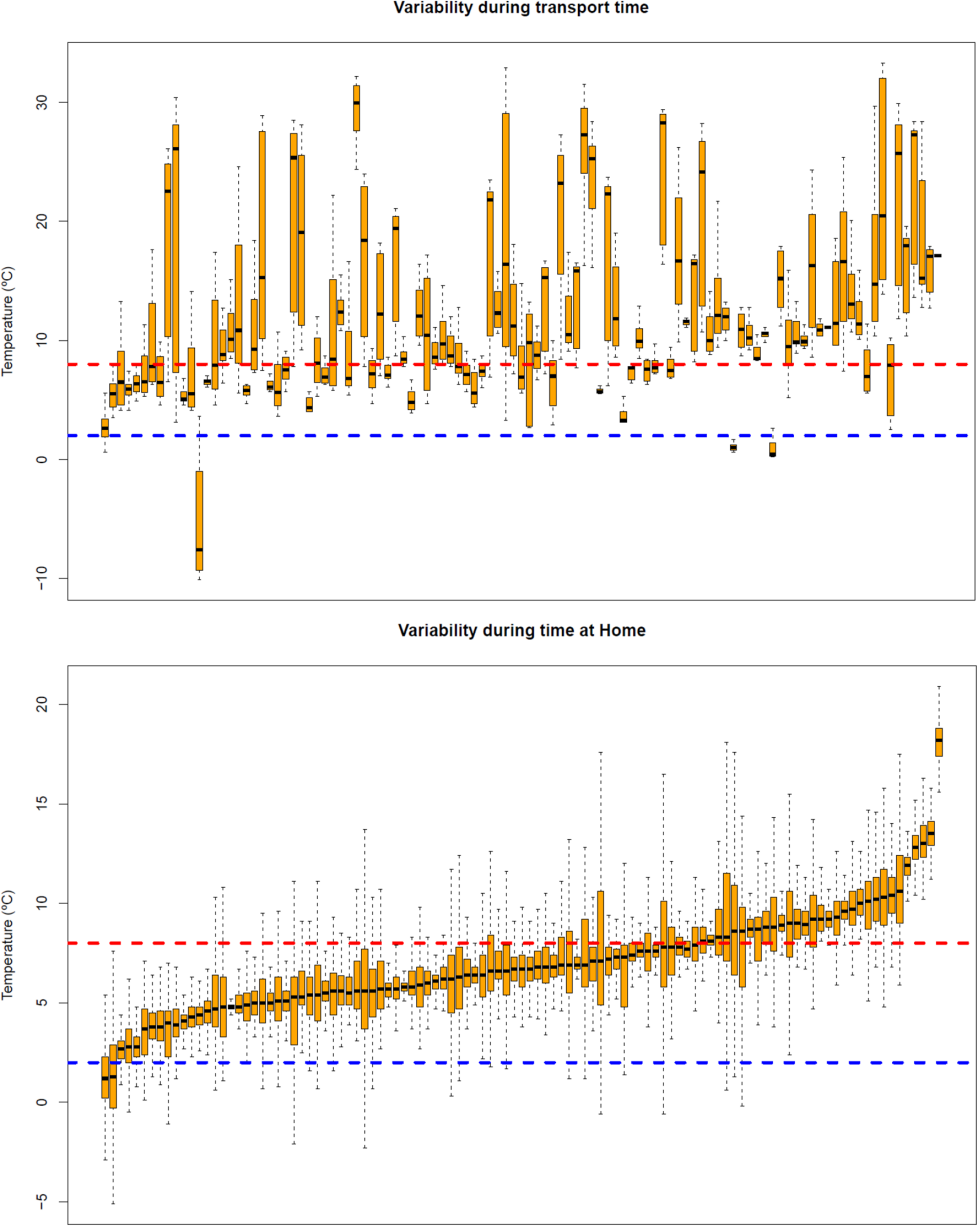
Box plot of each data logger during transport (**a**) and home storage (**b**). Listed according to median temperature during home storage. The whiskers represent Q3 + 1.5 × IQR to Q1 − 1.5 × IQR. Outliers are not shown due to the high volume of data.

The highest variability among data logger registers was detected during transport, with most mean temperatures out of range. During home storage, the majority of mean temperatures were within range, but with maximum and minimal measures outside the 2 °C to 8 °C range. Figure 3 represents the box plot of each data logger during transport and home storage.

**Figure 4:**
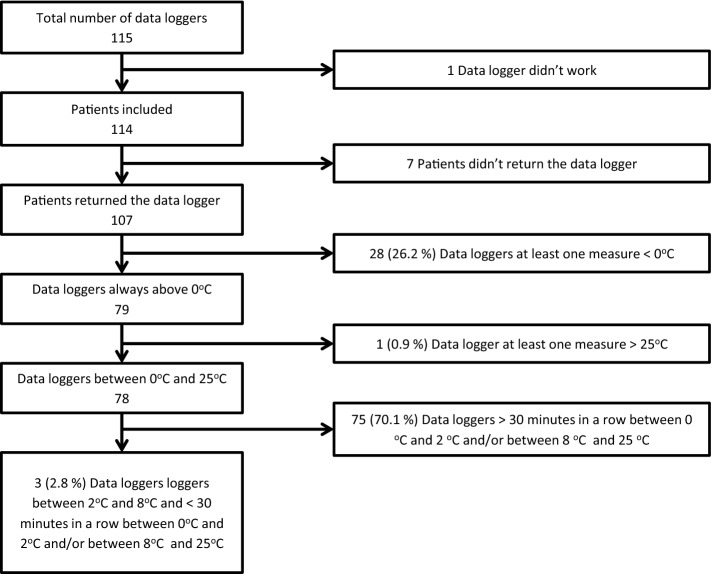
Flowchart of the data loggers included in the study showing home storage data.

Taking into account only the home storage period, according to our classification, 28 (26.2%) data loggers had at least one measurement below zero (Group 1). Among the rest, one data logger had one measurement greater than 25 °C (0.9%) (Group 2); most data loggers, 75 (70.1%), were between 0 and 2 °C and/or between 8 and 25 °C for more than 30 min (Group 3); in this Group, 50 (66.7%) data loggers were above 8 °C but not below 2 °C, 24 (32%) data loggers were below 2 °C and above 8 °C, and only in one case (1.3%) below 2 °C but not above 8 °C. Finally, only three data loggers (2.8%) maintained all the measurements between 2 and 8 °C with less than three continuous data readings outside this range but no data over 25 °C or less than or equal to 0 °C. Figure 4 represents the flowchart for the classification in different categories with home storage data.

Among the 28 data loggers with at least one measurement below zero, nine had a minimum temperature over − 1 °C and, among these, in seven cases the accumulated time in this category represented less than 100 min (0.16% of the time or less).

With respect to the 75 data loggers with all the measurements between 0 and 25 °C and over 30 min in a row between 0 and 2 °C and/or between 8C and 25 °C, 47 (62.7%) had a mean temperature within 2 °C and 8 °C, 8 (10.7%) had at least one measure below 1 °C and 55 loggers (73.3%) reached a maximum temperature over 12 °C. Seventeen (22.7%) loggers were always between 1 and 12 °C, with an accumulated time out of the range between 2 and 8 °C of less than 24 h (3% of the time or less) in eight cases.

## Discussion

We found that only 2.8% of thermolabile drugs were adequately stored in the ambulatory setting. This result is in line with some previous studies.

The first study confirming that in most cases thermolabile drugs were improperly stored once dispensed included 36 patients, finding that 50% of patients did not maintain a mean temperature between 2 and 8 °C during storage, while all drugs were out of range (mean temperature) during transport^3^. The same group published another paper focusing on the storage of two drugs (adalimumab and etanercept) among 60 patients, finding that 58.3% presented a mean temperature out of range. Refrigerators older than 5 years appeared as the only significant risk factor for inadequate storage. Both studies included a limited sample size and used mean temperature as the main variable^4^. Even though mean temperature was confirmed to correlate with consecutive time out of range in their second work, we believe mean temperature limits the results by reducing the amount of data. Moreover, their definition of improper storage was less strict than the present study.

A multicentre work in the Netherlands included nearly 300 patients and 750 data loggers, using a data logger for every package. They found that only 6.7% of the packages were stored between 2 and 8 °C with no excursions out of this range for more than 48 h in total or below 0 °C or above 25 °C for 2 h or more, continuously^5^. Out of 276 golimumab injectors, only 11.6% were adequately stored (no measurement below 0 °C and less than 30 min below 2 °C or over 8 °C) all the time in another study published in 2018. Mean storage time was 30.9 ± 33.1 days and 24.3% (67) injectors were below 2 °C > 30 min, and of these 46.3% (31) were below 0 °C > 30 min^6^. Although similar, our definition of correct storage is slightly stricter than in these studies, which may be the main reason why our results are comparable.

A recent study focused on insulin storage among 400 patients and found that 100% of the logs were out of the range from + 2 to + 8 °C at some point. The proportion of time out-of-range (11.3% with a CI 95%; 10.1% to 13.1%) was also measured, as well as the proportion of sensors that recorded temperatures below 0 °C (24.8%), which is very similar to our result (26.2%). However, in their study, patients had to purchase the sensors, which could have caused a selection bias^7^.

In all the above studies, it seems clear that domestic refrigerators do not keep the recommended temperature range. This question has also been studied in other fields such as food research, where a recent study conducted in our country showed that 17% of refrigerators had a mean temperature above + 8 °C or below + 2 °C^8^. Undoubtedly, this would be the main factor affecting the home storage of thermolabile drugs.

Compared to previous studies, we estimated sample size and included more drugs, but there are some possible limitations. Firstly, the study was limited to the home conservation phase, and the transport data of the medication was obviated. Secondly, for each patient, only one of the dispensed packages was measured, whereas the Vlieland^5^ and de Jong^6^ studies used a logger for each dispensed package, so they had more data. We assumed that storage conditions were similar for all the medications picked up at the same time, and instructed the patients to use the package with the logger last, in order to collect as much data as possible. Moreover, we did not assess whether there were any clinical consequences that could be associated with incorrect storage of medicines. It would be necessary to design another study focusing on clinical variables to evaluate this question.

We can assume that most thermolabile drugs are exposed to temperatures outside the range recommended in the Summary of Product Characteristics, but two main questions arise from this and previous studies. Firstly, we do not really know the clinical significance of this conclusion. In theory, temperature excursions may promote aggregate formation and could compromise drug activity and/or immunogenicity^9^, but though we found no paper dealing with clinical consequences in patients, Vlieland et al.^10^ studied aggregate and particle formation in four drugs included in the present work, concluding that stress conditions similar to daily practice had minor impact on these products. It would be of great interest to evaluate whether improper storage of thermolabile drugs may have any impact on medication effectiveness in daily practice; however, this would be questionable from the ethical point of view.

The second question is how to manage this situation. It seems obvious that it is necessary to improve transport conditions of drugs and patient training. Apart from technology like cooler bags or data loggers, we believe that educational materials for the patients to become aware of the importance and potentially effects in case of improperly storage, like a lack of efficacy or adverse events, could be useful. On the other hand, smaller quantities of medication could be dispensed at a time to ensure that it is properly stored in the Pharmacy Department; however, this would cause a tremendous increase in patient visits to the Hospital Pharmacy. The thermolabile drug could also be sent to the patients’ home just before they need it, but our health system is not yet prepared to take on this possibility. Another option would be to create an alarm system with this type of medication to notify the patient in case of loss of efficacy due to temperature excursions, similar to operating procedures used in daily practice at Pharmacy Departments in the Event of Cold Chain Failures^11^.

In conclusion, most thermolabile drugs are improperly stored at patients’ home. Future studies should focus on clinical consequences and possible solutions.

## Methods

### Design

A prospective observational study was performed to assess the temperature profile of thermolabile drugs once dispensed to ambulatory patients at a tertiary care hospital.

A continuous temperature measurement system was used to record thermal conditions from dispensation until the next outpatient visit.

### Setting and participants

Son Espases University Hospital is a tertiary hospital located in Palma de Mallorca in the Balearic Islands, Spain. It is the reference hospital of a region with a population of more than one million inhabitants. The Pharmacy Department dispenses medications to over 7000 patients every year. Patients were included in the study from July 2018 to January 2020.

### Thermolabile medications

The following seven widely dispensed thermolabile medications were selected for the study in order to guarantee the number of patients needed and to avoid paediatric population and blood products: adalimumab, certolizumab pegol, darbepoetin alfa, erythropoietin, etanercept, trametinib, and peginterferon alfa 2-a.

### Inclusion criteria

1. Outpatients with a prescription of any of the selected thermolabile medications dispensed at the Pharmacy Department of Son Espases University Hospital.
2. Signature of written informed consent.
3. Older than 18 years.

### Exclusion criteria

1. Patients who do not give their consent.
2. Patients participating in a clinical trial.
3. Patients who presented some type of inability to understand the development of the study, such as a language barrier or intellectual disability.
4. Patients who lived in residences and/or were admitted to nursing homes.

### Intervention

Patients were recruited in the Pharmacy Department at the time of dispensing the thermolabile medication. When the patient came to the Pharmacy Department to pick up any of the medications included in the study, they were informed and offered the opportunity to participate. If the patient agreed, a questionnaire regarding the conditions that may affect the preservation of thermolabile drugs was completed.

Along with the medication, a data logger was added to the medication packaging. The data logger is a temperature recorder programmed to perform periodic measurements during the period between visits to the Hospital Pharmacy. If the patient was given more than one box, the container with the data logger was the last to be administered. The patient had to return the medication packaging and the data logger to the Pharmacy Department in the next visit, and the information was downloaded to analyse the data recorded. One medication was monitored for each patient. In case of detecting inadequate preservation, the importance of maintaining the medications in accordance with the recommendations of the Summary of Product Characteristics was emphasized.

### Data logger characteristics

Temperature sensors TempTale 4USB, Sensitech INC, MA USA were used. Accuracy was ± 0.05 °C and calibration range was from − 195 to 232 °C. Once started, temperature was recorded every 10 min.

### Outcomes

The primary outcome was the proportion of TD improperly stored in the patients’ home setting. Temperature was considered inappropriate if one of the following circumstances were met: any temperature record less than or equal to 0 °C or over 25 °C; temperatures between 0–2 or 8–25 °C for a continuous period over 30 min (three continuous measures).

Secondary outcomes included: proportion of TD with at least one temperature value less than or equal to 0 °C, proportion of TD with at least one temperature value over 25 °C, total time TD had been less than or equal to 0 °C, total time TD had been over 25 °C, total time TD had been within 8–25 °C, and total time TD had been over 8 °C.

As transport is the most sensitive period to temperature changes, we divided the data set into transport and home storage to analyse the thermal profile. The transport period was defined between the first temperature sample and the moment when the medication was placed in the refrigerator at home. The division between periods is based on a time series analysis algorithm defined by Bai^12^. The algorithm was implemented in R language by Marc Lavielle in 2017 and used in this study to find tendency changes in thermal profile^13^. This algorithm was applied using different considerations: first, considering the refrigerator works properly, therefore the home storage period will produce a smoother, less variable thermal profile than during the transport period. Second, related to the maximum transport period duration, since the patient is expected to store the medicine in the refrigerator within the first 24 h from the time it was picked up. In Lavielle (2017), the mathematical model of the algorithm applied assumes that change point is the time point when the least-square method minimizes the residual sum of squares. Hence, the algorithm implemented splits the time series between transport and home storage periods where the residuals sum of squares of each period is minimum. The complete detail of this mathematical procedure can be found in Lavielle^13^.

Additionally, the research team added 1 h to the instant time obtained by the algorithm in order to include in the transport period the time needed by the medicine to reach the refrigerator temperature. Consequently, the transport period started when the patient received the medicine and the data logger was started, and ended when the algorithm detected the change point in thermal profile plus one hour more.

Medications were categorized sequentially into four exclusive groups:

- *Group 1* TD has at least one measure less than or equal to 0 °C.
- *Group 2* TD has at least one measure over 25 °C.
- *Group 3* TD has at least three continuous measures between 0 and 2 °C or between 8 and 25 °C.
- *Group 4* All other medications considered to be properly stored: measures between 2 and 8 °C with less than 3 continuous data out of this range but no data over 25 °C or less than or equal to 0 °C.

### Sample size

Drugs dispensed to outpatients at the Pharmacy Department were analysed over 6 months, and 1403 patients picked up TD, 625 of which were medications included in this study. Based on data in the literature, it was difficult to estimate the proportion of TD improperly stored, so we considered 50% to maximize the population.

A sample size of 107 randomly selected subjects was sufficient to estimate with 95% confidence and a precision of ± 10 percent units, a proportion of TD improperly stored considered to be around 50%. A replacement rate of 25% was anticipated.

### Data analysis and statistics

The recorded data were analyzed using R software version 3.6.3 on a Linux platform with the RSTUDIO IDE as user interface on real thermal data. The time series of temperature measurements obtained from each data logger were analyzed as statistically independent variables as each data logger was attached to different medicines and stored in different refrigerators and the total period of measurements were not the same in all cases, in some cases during winter and other cases during summer.

The data shown in the results section did not undergo any statistical treatment and must be considered directly related to thermal measurements.

### Ethics approval

This study was approved by the Ethics Committee of the Balearic Islands. Reference Number IB 3693/18 PI. All the interventions were performed in accordance with relevant guidelines and regulations.

## Data Availability

The datasets used and/or analysed during the current study are available from the corresponding author on reasonable request.
